# Food Integrity Climate in Food Businesses: Conceptualization, Development, and Validation of a Self-Assessment Tool

**DOI:** 10.3390/foods10061302

**Published:** 2021-06-06

**Authors:** Waeel Salih Alrobaish, Liesbeth Jacxsens, Pieternel A Luning, Peter Vlerick

**Affiliations:** 1 Department of Food Technology, Safety and Health, Faculty of Bioscience Engineering, Ghent University, Coupure Links 653, 9000 Ghent, Belgium; liesbeth.jacxsens@ugent.be; 2 Food Quality and Design Group, Wageningen University and Research, P.O. Box 17/Bode 30, 6700 AA Wageningen, The Netherlands; pieternel.luning@wur.nl; 3 Department of Work, Organization and Society, Faculty of Psychology and Educational Sciences, Ghent University, Henri Dunantlaan 2, 9000 Ghent, Belgium; peter.vlerick@UGent.be

**Keywords:** food integrity, food integrity climate, food safety climate, self-assessment tool

## Abstract

Current scientific research and industry guidelines focus on food safety, aiming to reduce unintentional food contaminations through technological and managerial measures. Due to the deceptive nature of food fraud, the fight to prevent intentional food adulteration and counterfeiting threats requires an approach that goes beyond the common food safety-based strategies and falls into the sphere of food integrity. With food integrity being an emerging discipline, a definition was proposed and the concept of food integrity climate was introduced. A food integrity climate (FIC) self-assessment tool in the form of a questionnaire, with twenty indicators and a five-point Likert rating scale was developed, expert-validated, and tested in practice in a large-scale meat distribution company. The questionnaire was designed to measure the performance level of food integrity in food businesses along the supply chain through managers’ and operators’ perceptions. Minor but interesting differences were found in the food integrity climate perceived between managers and operators as well as among the company’s affiliates. The tool helps food businesses to get a deeper insight on the human dimension behind food integrity through the assessment of five climate components in relation to four food integrity elements, identifying strengths and weaknesses regarding a company’s food integrity climate.

## 1. Introduction

Scientific research, industry standards, and (inter)national regulations focus primarily on food safety, aiming to reduce unintentional food contaminations through analytical methods, technological interventions, implementation of food safety management systems, certifications, and audits [1,2,3]. These measures are not specifically designed for food fraud control, mitigation, and prevention, since they do not consider the intentional contamination and economically motivated driven behavior leading to unacceptable risks [4,5,6]. Due to the intentional and deceptive nature of food fraud, the fight to reduce and prevent deliberate food adulteration and counterfeiting threats requires an approach that goes beyond the common food safety-based strategies and falls into the sphere of food integrity [7,8,9,10].

The concept of food integrity goes beyond the mere food safety-related concerns, comprising in its notion all the aspects of food processing, handling, and monitoring occurring along the food supply chain. As two opposed faces of the same coin, food integrity and food fraud consider not only technical and managerial factors but also the human dimension involved in the actual execution of processes [5,8,11].

Few scientific studies have been conducted on the theoretical conceptualization of food integrity and its defining elements [5,8,11,12,13,14,15]. However, as food integrity is an emerging discipline, its concept remains still unclear and no unanimous definition exists yet. A deeper insight into the specific role of the human factor in food integrity still needs to be addressed. Recent food safety research revealed that the techno-managerial approach towards food safety should be complemented by a human route, as expressed by operators’ perceptions of the organizations’ food safety climate [16]. Manning (2020) [10] also advocates the importance and the need of developing tools to assist food companies to evolve from a compliance-based organizational food safety climate to an ethically strong organizational climate that focuses on the more comprehensive concept of integrity. Moreover, Ling and Wahab (2020) [6] argue that the implementation of food safety management systems based on prerequisites programs and HACCP principles alone is insufficient since it does not prevent deliberate contamination as it is designed for food safety hazard control rather than food integrity.

This paper proposes a definition of food integrity and, in consideration of the human dimension involved within the organizations’ decision-making processes, introduces the concept of a food integrity climate. The development, expert validation, and testing in practice of a food integrity climate (FIC) self-assessment tool, designed to specifically measure the food integrity performance level in food companies along the supply chain through managers’ and operators’ perceptions, is detailed. Implications concerning food integrity management for food businesses are derived from the application of the FIC tool in a large-scale meat distribution company.

## 2. Materials and Methods

### 2.1. Defining Food Integrity

Based on a literature study on the domain of food integrity and related concepts, such as food safety, food quality, food authenticity, food defense and food fraud, as well as discussion with three subject-matter academic experts operating in Belgium and the Netherlands (an expert on food safety management, an expert on work and occupational health psychology, and an expert on food quality and design), a definition of food integrity is proposed and a schematic representation of the nature and interconnection of food integrity and food fraud elements is presented.

### 2.2. Conceptualizing Food Integrity Climate

Based on a literature study in the domain of organizational climate and food integrity, as well as discussion with three subject-matter academic experts operating in Belgium and the Netherlands (an expert on food safety management, an expert on work and occupational health psychology, and an expert on food quality and design), the concept of a food integrity climate is introduced along with its definition and key elements. A conceptual model of food integrity climate is also presented.

### 2.3. Development of a Food Integrity Climate Self-Assessment Tool

Based on the proposed food integrity climate definition and elements, a self-assessment tool was developed to assess the food integrity performance level as perceived by employees of food companies along the supply chain. Indicators were formulated based on literature reviews on relevant subjects such as food fraud, food safety climate and food integrity, as well as following discussion with three subject-matter academic experts operating in Belgium and the Netherlands (an expert on food safety management, an expert on work and occupational health psychology, and an expert on food quality and design).

### 2.4. Expert Validation of the Food Integrity Climate Self-Assessment Tool

To examine the validity of the developed FIC tool, a balanced panel of twelve experts, not involved in the development of the tool, with expertise in food safety and quality management at the academic level (six experts) and at the industry level (six experts), operating in Belgium, Italy, and Saudi Arabia, were asked through an online survey to evaluate the relevance and the importance of each indicator of the tool. The academic experts were selected based on their knowledge in constructing self-assessment tools for the evaluation of food quality, food authenticity, and food safety management systems within food organizations, while the industry experts were chosen based on their experience in managing food safety and food integrity-related issues in food companies.

Inspired by the method adopted by De Boeck et al. (2015) [16], each expert had to indicate whether they considered the twenty indicators of the preliminary version of the tool relevant (“Does the indicator add to the understanding of food integrity climate?”) by means of a yes/no answer. Further, they had to rate the importance or validity of each indicator (“Does the indicator measure an important aspect of food integrity climate?”) by means of a four-point Likert answer scale from very important (1), important (2), somewhat important (3), to not important (4). Experts were also given the possibility to write open suggestions to improve the comprehensibility of each indicator.

If 50% or less of the responding experts did not consider the indicator relevant, this was deleted. For the importance rating, the median, mean, and standard deviation of each indicator were calculated. Indicators were considered valid if they were rated overall from very important (1) to somewhat important (3). Finally, based on the open suggestions, indicators could be made more understandable and meaningful, or new indicators could be added.

### 2.5. Testing in Practice of the Food Integrity Climate Self-Assessment Tool

To test the feasibility of the FIC tool when applied in a food business context, a single testing of the FIC tool was performed in eight randomly selected affiliates of a large-scale meat distribution company with over three hundred branches and almost four thousand employees in Belgium and Luxembourg. All operators (*n* = 34) in the eight affiliates, including chefs, assistants, and salesmen, and their management (*n* = 18), including general director, quality manager, sales managers, and managers in charge of the affiliates, were invited to participate in the study. The researchers personally handed out a printed version of the FIC tool in Dutch to the participants. A cover letter was attached to the questionnaire explaining the scientific purpose of the study and stated that, although participation was not mandatory, filling in the survey implied consent and that confidentiality was guaranteed. The researchers’ contact details were also provided. Upon filling in, the participants were asked to send back the questionnaires using the internal post service. Informed consent was obtained from the board of directors of the participating company to conduct the study and publish anonymously the results.

Inspired by the method adopted by De Boeck et al. (2015) [16], the research objectives of the study were to (1) assess the company’s food integrity climate, (2) compare how this is perceived among the eight different affiliates, (3) evaluate if there is a difference in perception between management and operators, and (4) estimate statistically the reliability of the tool. To achieve the first three research objectives, the mean score of the overall food integrity climate (calculated over the twenty indicators), the mean score of each food integrity element (calculated over the five indicators per element), the mean score of each climate component (calculated over the four indicators per component), and the mean score of each food integrity climate indicator was calculated for the total sample, for the management, each affiliated butcher shop, and all affiliates’ operators. Finally, the Cronbach’s alpha of the questionnaire was calculated to assess the reliability and internal consistency of the tool. Data processing was executed using IBM SPSS version 26.

## 3. Results and Discussion

### 3.1. Defining Food Integrity

Food integrity has been defined by Elliott (2014) [13] as the condition of a food product to be safe, of quality, authentic, traceable, and genuine in all its aspects, whose nature has not been altered or modified and whose claims are honest and meet consumer expectations. Accordingly, it is agreed that food integrity is a multidisciplinary and multidimensional concept, covering all aspects of the food chain, from producer to consumer, and capturing all aspects of food production, such as the way the food has been sourced, produced, and distributed [6,12,13,17].

**Figure 1:**
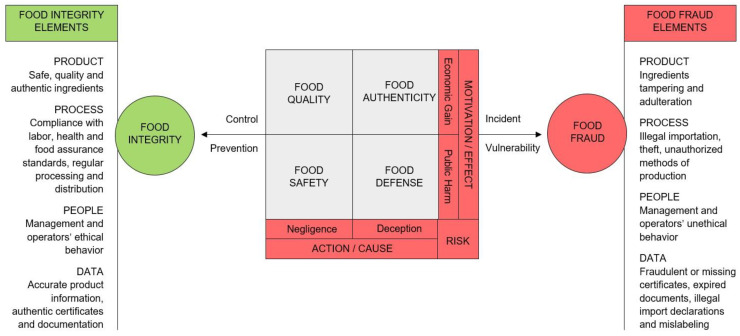
Schematic representation of the nature and interconnection of food integrity and food fraud elements. As two opposed faces of the same coin, food integrity and food fraud both concern the entirety of the food supply chain aspects involved in the food production, including product, process, people, and data, as well as the activities employed in the monitoring of food, such as quality, safety, authenticity, and defense. In the case of food fraud, such elements and activities are managed fraudulently and improperly causing incidents and vulnerability along the food supply chain. Oppositely, food integrity represents the ethical manner and preventive approach through which food companies control the overall food production (adapted from Spink and Moyer (2011) [24] and Manning (2016) [8]).

Manning (2016) [8] argued that food integrity includes four elements that need to be considered to safeguard integrity in the food supply chain: product integrity, process integrity, people integrity, and data integrity, defined in Figure 1 (left side).

Similarly, Ali et al. (2017) [15] suggested that food integrity comprehends the whole food supply chain, involving particularly four distinctive dimensions: (1) raw material integrity, at the supplier stage, meaning safe, pure, quality ingredients with authentic and verifiable origins; (2) production integrity, at the manufacturer stage, referring to effective internal control system, quality assurance, manufacturing strategies, and procedures; (3) service integrity, at the food service stage, regarding controlled franchises, outsourcing activities and human resources involved by a food company as third party operators; and (4) information integrity, at the consumer stage, concerning truthful, honest, and appropriate data conveyed to the consumers through the product labeling and company logo.

Other attributes recurring in most food integrity definitions relate to healthy, tasty, and nutritional product ingredients [5,12] that are sustainable in nature, considerate of animal welfare, free of child labor, and favor trades with developing countries [12]. This creates consumer trust and brand integrity [14], by safeguarding the food supply chain from fraud risks [11] and considering cultural matters in the processing and handling of food [5]. It can be deduced that food integrity refers not only to the safety [18] and quality [19,20] of a food product but also to its authenticity [13] as well as its defense or protection from unintentional contamination hazards and deliberate counterfeiting threats [17,21,22,23].

While food quality and food safety hazards occur mostly unintentionally or as a consequence of negligent handling of food and limited control during the production stages, food authenticity and food defense threats are intentionally perpetrated to deceive consumers through mislabeling and counterfeiting (Figure 1). Food quality and food authenticity incidents affect primarily the economic sphere, whereas food safety and food defense risks may severely impact public health [24].

Food integrity comprises in its notion all the properties of a food product included in the common definitions of food safety, quality, authenticity, and defense [6]. These concepts are, in fact, product-centric referring to one or more aspects of food, such as ingredients, production methods, and supportive documentation, whereas the notion of food integrity overarches them all and is system-centric [11]. Therefore, the relation between food integrity and food fraud is inversely proportional, as also demonstrated by Manning (2016) [8], who explains food fraud as the result of a misrepresentation associated with product, process, people, and data integrity, as detailed in Figure 1 (right side). When all aspects related to food quality, safety, authenticity, and defense are kept under control through efficient risk assessment, management, and prevention systems, food integrity may be achieved. Contrarily, companies will be vulnerable to food fraud.

Based on all the arguments above, aiming to achieve a unanimous definition, we define food integrity as “a multidimensional concept concerning the integrity of product, process, people, and data, implying the controlled status of a food product to be intact, safe, of quality, and authentic in its claims, as well as sourced, processed, and distributed ethically throughout a food supply chain.”

This definition aligns with the general concept of integrity described by Jacobs (2004) [25], who explains integrity as existing within two categories: physical and moral integrity. In the first conception, integrity applies to the physical state of undivided wholeness, while in the latter, it connotes an unimpaired moral state, characterized by innocence, uprightness, honesty, and sincerity, mostly in the context of business ethics.

### 3.2. Conceptualizing Food Integrity Climate

In a business environment, organizational climate refers to the shared perceptions and the meaning attached to the policies, practices, and procedures employees experience at work and the behaviors they observe that are supported and expected [26]. It originates from individual perceptions derived from workers making sense of their work environment that become shared when they converge in the same organization [27]. Previous studies identified five key types of perceptions, such as leadership, communication, commitment, risk awareness, and resources as fundamental human aspects of a company’s climate [28,29,30,31,32,33,34,35].

The relevance of these five human drivers of a company’s climate has been demonstrated in the context of food safety [16] where a positive relationship between the food safety climate as perceived by employees and their food-related behavior was found. A similar relationship and the role of these five climate components was replicated in the endovascular surgery field, showing a strong positive direct and indirect effect of the perceived radiation safety climate on radiation safety behaviors of team members [36].

**Figure 2:**
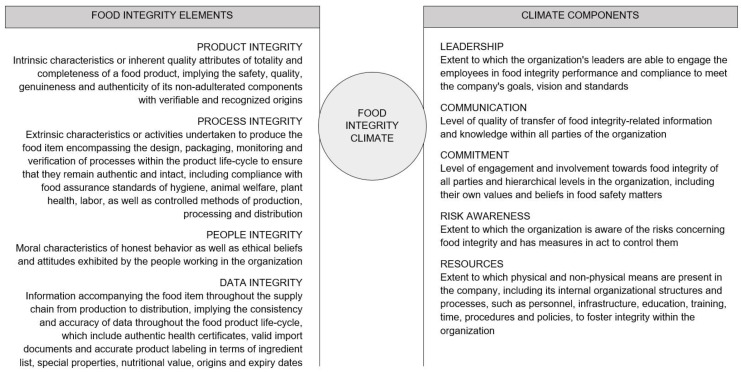
Conceptual model of the food integrity climate. The concept of a food integrity climate is constituted by the combination of food integrity elements (product, process, people, and data integrity) [8] and the components of a company’s climate (leadership, communication, commitment, risk awareness, and resources) [16].

These human or climate components remain unstudied in the domain of food integrity. As it is assumed that the five climate components are also relevant in the context of food integrity, they were integrated with the four food integrity elements (product, process, people, and data integrity) distinguished by Manning (2016) [8], defined in Figure 2, for the elaboration of the food integrity climate definition.

We define food integrity climate as “the employees’ shared perception of leadership, communication, commitment, risk awareness, and resources regarding food integrity within the company’s working environment in terms of product, process, people, and data integrity.”

### 3.3. Development of the Food Integrity Climate Self-Assessment Tool

Based on the proposed food integrity climate definition and elements, a self-assessment tool was developed to assess the food integrity performance level as perceived by employees of food companies along the supply chain. As the overall aim was to capture human perceptions, a self-report type of measure was adopted in the form of a questionnaire that employees could fill out. This choice is in line with the widespread use of self-reported scales, where self-assessment surveys have been used to investigate employees’ perceptions, knowledge, and behavior regarding a variety of organizational issues, even in the fields of food safety [16,37,38,39] and food fraud [4].

The FIC tool was structured with four sections, one for each food integrity element. For each section, five indicators were developed, one for each of the five key climate components. The twenty resulting indicators, or questions in the form of statements, were elaborated content-wise by combining the defining attributes of the four food integrity elements with those of the five climate components, adapted to suit food integrity-related matters, as shown in Table 1.

To facilitate the comprehension of the questionnaire indicators, concrete situational examples were added to each statement. Definitions of food integrity and its elements were given at the beginning of each section in a user-friendly language. To avoid response biases by participants, such as acquiescence and social desirability, which can be a drawback with self-report measures, and to assure content validity, sixteen positively formulated items were mixed with four negative or reversed items (one for each section: Q2, Q10, Q13, and Q17) [40,41,42,43].

Since the goal of the tool was to investigate the food integrity climate from employees’ point of view, a five-point Likert response scale was adopted [16,44,45,46,47,48]. Respondents were asked to evaluate each indicator from 1 to 5, corresponding to strongly disagree (1), disagree (2), not agree nor disagree (3), agree (4), and strongly agree (5), where responses closer to 5 imply a better-perceived food integrity climate depending on the particular indicator. The answer scale of the four negatively formulated indicators was afterward recoded in reverse order during the data processing. The final version of the FIC tool is reported in Appendix A.

#### 3.3.1. Indicator Rationale for the Food Integrity Climate Element: Product

Setting clear objectives in an organization enables the measurement of performance against the defined targets, which represents a first critical step for the continuous improvement towards food safety and, by extension, food integrity [29]. Good management within an organization involves planning, organizing, leading, and controlling goal-oriented activities. Leaders should coordinate and oversee the work of the employees so that the activities are completed efficiently and effectively [49]. As such, statement Q1 was introduced in the questionnaire (see Table 1).

Regular and clear social exchanges between leaders and employees can lead to better communication concerning food integrity matters. Leaders should communicate often and plainly with their employees to ensure that employees are aware of their roles and responsibilities [29]. Thus, Q2 was introduced.

Leaders must demonstrate that achieving food integrity is more important than productivity and profit. If employees’ own beliefs and values align with those of the organization, they will be more motivated to achieve food integrity and exceed expectations, as they will not see it merely as their task, but believe in it [31,32]. Leaders with integrity sell more effectively because they seek to sell genuinely valuable products [50]. Therefore, Q3 was introduced.

Risk perception and the subsequent risk-taking behavior are critical for achieving a good food safety climate and, in general, a good food integrity climate. Risks should be known by the employees, so they can take them into account in their daily work decisions and avoid incidents [31]. To prevent food supply chain fraud, leaders must understand the factors that enable the occurrence of risks, such as criminal actions able to deceive existing scientific testing processes [1]. Hence, Q4 was introduced.

If employees are given sufficient time to work in a way that will ensure the achievement of food integrity, they will likely perceive a high psychological and emotional support from their organization. High perceived organizational support also implies that sufficient staff are available to follow up and collaborate on daily working activities, so that every staff member may have sufficient time to work efficiently to achieve food integrity [31]. Furthermore, measures such as contractual requirements with suppliers addressing specifically fraud prevention, extensive self-regulation and social control across the supply chain, transparent feedback, and actions on criminal behavior to enhance fraud awareness and support fraud mitigation measures can contribute to the reduction of fraud vulnerability [4]. Accordingly, Q5 was introduced.

#### 3.3.2. Indicator Rationale for the Food Integrity Climate Element: Process

Efficient leaders set clear expectations for the employees so that all the employees in the organization know what is expected from each one of them and what they are required to do to meet such expectations [29]. As such, Q6 was introduced in the questionnaire.

Appropriate communication and language should be used and additional effort should be spent to ensure that food integrity-related messages are clear to employees who do not always share the same language as the leaders [33]. Thus, Q7 was introduced.

Leaders should be committed to ensuring food integrity, and consider this a priority by responding quickly and actively to solve potential problems. Remarks concerning food integrity issues should be addressed constructively and respectfully to avoid a blame culture, in which employees are discouraged to admit their mistakes because of possible negative consequences [30,31]. Employees with high integrity are more rational, honest, independent, as well as innovative and productive [51]. Furthermore, leader behavioral integrity can improve error reporting and error management within the organization, leading to a reduction in malpractices and food fraud risks [52]. Contingency plans should also be present to deal with potential emergencies and to ensure that incidents do not escalate [4]. Accordingly, Q8 was introduced.

It is important for leaders and employees to feel they can trust and rely on the performance of the organization’s food safety management systems and food fraud prevention strategies. Such trust can motivate employees to remain committed toward the achievement and improvement of food integrity [35]. Trust is needed within the organization and along the whole food supply chain, and such trust can only be created and maintained if promises are kept and guaranteed by effective and efficient controls [14]. Therefore, Q9 was introduced.

Organizational support is reflected by the availability of the necessary infrastructure, such as proper equipment and workspaces, and sufficient technical and financial resources to assist in the achievement of food integrity [29]. Monitoring systems to supervise processes and control fraud are essential tools for organizations and should be in place to evaluate, remedy, and improve food fraud prevention and detection techniques [4]. Hence, Q10 was introduced.

#### 3.3.3. Indicator Rationale for the Food Integrity Climate Element: People

As employees are the frontline workers on the organization’s work floor, they are often the first to notice deviations and expose issues and opportunities on food integrity matters. Therefore, it is important and beneficial for leaders to listen to employees’ comments and suggestions [29]. If employees recognize that they have a critical role in the achievement of food integrity, they will also feel proud about the positive results of the organization [30,31]. From the perspective of integrity, the task of leadership is to define and give life to an organization’s guiding values, to create an environment that supports ethical behavior, and to instill a sense of shared accountability among employees [53]. As such, Q11 was introduced in the questionnaire.

Employees should feel free to approach colleagues who are engaged in behavior that can be potentially harmful and to openly discuss with leaders about food integrity issues within their organization. This will contribute to the openness and honesty within the organization and may be beneficial for the food integrity climate [28,31]. When leaders are not open and honest at all times, employees will feel that they can get away with lying occasionally, including deceiving supervisors or falsifying reports and records. Thus, Q12 was introduced.

Consequences determine whether a behavior is repeated. Positive consequences stimulate good behavior, while negative ones are less effective in influencing long-term behavioral change, working rather on the short term [29]. Leaders should motivate their employees, give positive feedback and acknowledge good behavior [31]. A culture characterized by demotivation, mistrust, and dissatisfaction can be a breeding ground for unethical behaviors among employees. Unethical business cultures lead to the normalization of committing food fraud, which reinforces longer-term fraudulent activity within the organization and supply chain [4]. Therefore, Q13 was introduced.

Employees’ trust and positive commitment to the organization’s systems and strategies can be enhanced if employees perceive that their colleagues and leaders are alert, attentive, and carefully considering food integrity issues in the right manner. If employees think that leaders overestimate or underestimate risks, they will be less inclined to strive to achieve high levels of food integrity [31]. Leaders with moral integrity do not justify low wages and poor working conditions by their firms’ need to be competitive and profitable [25]. Hence, Q14 was introduced.

Many integrity initiatives have structural features common to compliance-based initiatives: a code of conduct, training in relevant areas of law, mechanisms for reporting and investigating potential misconduct, and audits and controls to ensure that laws and company standards are met [53]. Frequent education and training concerning food integrity are needed to achieve behavioral change [29]. Ethical codes of conduct consist of moral standards which help to guide employee or corporate behavior. Codes of conduct are favorable toward the creation of a stronger ethical corporate climate and relate to positive ethical behavior, reducing food fraud vulnerability [4]. Accordingly, Q15 was introduced.

#### 3.3.4. Indicator Rationale for the Food Integrity Climate Element: Data

Leaders striving for continuous improvement often denote strong leader ambition and reflect the importance of working to achieve food integrity within the organization and outside [16]. With regard to data integrity, the striving for continuous improvement within a food organization implies being honest and correct about product information. As such, Q16 was introduced in the questionnaire.

If employees are constantly reminded of the importance of food integrity through clear communication, they will be more inclined to adopt this belief. The use of various mediums to convey food integrity-related messages can increase effectiveness [29]. Accordingly, Q17 was introduced.

Leaders should set a good example concerning food integrity since the actions of the leaders will be adopted by the employees. If leaders participate in the critical everyday tasks, demonstrating the importance of food integrity, employees will pay more attention to safety and integrity matters themselves [31]. Leaders should serve as role models by following proper food integrity practices themselves. Leader behavioral integrity is, in fact, critical for shaping employee attitudes [52]. Creating an organization that encourages exemplary conduct may be the best way to prevent damaging misconduct [53]. Hence, Q18 was introduced.

Effective risk communication is essential to ensure that both leaders and employees possess a realistic picture of the potential risks concerning food integrity in the organization and act accordingly [31]. Leaders’ risk communication is an effective technique to achieve behavioral change since it can positively influence employees’ risk awareness and how employees will act in matters related to food integrity [34]. Unless food supply chains take the holistic view of food fraud and mitigation strategies to the core of their operations, the criminal elements will always have a gap in the chain that can be exploited [54]. Therefore, Q19 was introduced.

Effective procedures and instructions concerning food integrity-related matters should be constantly reminded, written down, and documented within the organization to ensure that employees clearly know what is expected from them to prevent deviations and doubts about specific processes and activities [29]. Furthermore, the use of indirect data, such as those from tracking and tracing systems can contribute to fraud control. Due to the ability to find information on the history, process, as well as the location of a product or ingredient, traceability tools can be used to prevent or eliminate illegal, unreported, and unregulated products [4]. Thus, Q20 was introduced.

### 3.4. Expert Validation of the Food Integrity Climate Self-Assessment Tool

Results of the relevance evaluation and the importance rating are reported in Table 2. All indicators were considered relevant by more than 50% of the experts. Therefore, none of the twenty indicators were considered for deletion. Based on the median calculation of the importance ratings, the tool could be considered valid, as all the indicators were rated overall from very important (1) to important (2).

No new indicator was added to the questionnaire based on the information provided by the experts in the open suggestions. However, some minor lexical modifications and textual adjustments were made to improve the clarity of a few statements. The final version of the indicators and the tool are reported respectively in Table 1 and Appendix A.

### 3.5. Testing in Practice of the Food Integrity Climate Self-Assessment Tool

Statistical exploration of the data showed remarkable findings. Firstly, the company’s overall food integrity climate as perceived by all the participating employees (managers and operators) (*n* = 52) was high (mean = 86.73/100). The total sample perceived the product integrity to be the best-performing food integrity element (mean = 22.10/25), while the people integrity was the lowest-performing one (mean = 21.19/25). With regard to the climate components, the best performing component was perceived to be the leadership (mean = 17.71/20), while the worst performing was the one related to the company’s resources (mean = 16.92/20).

Secondly, statistical differences among the eight affiliates were found regarding the perceived food integrity climate. Affiliates could be clustered in three different groups: (1) three affiliates evaluated the food integrity climate as very high (means range = 90–100/100), (2) four different affiliates rated it as high (means range = 80–90/100), and (3) the remaining affiliate scored it as medium (mean range = 70–80/100; mean = 70.67/100). Even though the results are not representative of the full organization (only eight affiliates from the over three hundred butcher shops were involved), it could be noted that the two best scoring affiliates perceived the data integrity as the best-performing food integrity element (mean = 24.67/25), while the worst scoring affiliate evaluated people integrity as the lowest-performing food integrity element (mean = 16.00/25).

Thirdly, no significant statistical differences were found in the mean scores for the overall food integrity climate nor in the mean scores for the four food integrity elements separately regarding the perceptions between operators and their managers in comparison. Both subsamples perceive a similar high food integrity climate (management mean = 87.22/100; operators mean = 86.47/100).

Concerning the four food integrity elements, the management rated product and people integrity slightly higher than the operators, while the operators perceived process and data integrity slightly higher than the management, but not significantly different. This may be due to their different organizational roles and tasks. For similar potential reasons, the management estimated the product integrity as the best-performing food integrity element and the process integrity as the worst-performing one. On the other side, operators scored the process integrity as the best-performing food integrity element and the people integrity as the worst-performing one. Mean scores and *p*-values are reported in Table A1 in Appendix B.

Of the five climate components, the company’s resources was the component perceived most dissimilarly by the two sample groups, with the management rating this component higher than operators (management mean = 17.89/20; operators mean = 16.41/20). This can be explained by the fact that, in the field, tangible and usable things such as the company’s resources (e.g., production materials, databases, workspaces, codes of conduct, employees’ training, and services) are acknowledged differently by those who remotely administrate them (managers) and those who concretely utilize them in the everyday working practices (operators). No meaningful statistical differences between operators and their managers were found for the other climate components, which are immaterial and abstract dimensions.

**Figure 3:**
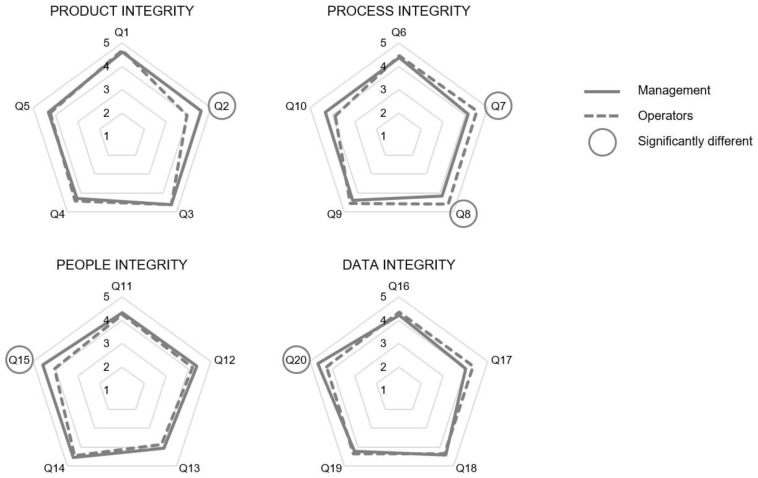
Radar chart with mean results of management (*n* = 18) and operators (*n* = 34) of the participating large-scale meat distribution company on the indicators of the food integrity elements. A five-point Likert answer scale (1→5: strongly disagree→strongly agree) was used. The indicators that showed a statistically significant difference between management and operators’ scores are circled (*p* < 0.05). (See mean scores and *p*-values in Table A1 in Appendix B).

A more detailed analysis at each single indicator level revealed statistical differences between management and operators in five indicators (Q2, Q7, Q8, Q15, and Q20), as shown in Table A1 in Appendix B and represented in Figure 3. Managers scored higher than operators in Q2, Q15, and Q20, where two of these indicators (Q15 and Q20) are related to the climate component resources regarding respectively people and data integrity, while Q2 concerns the component communication in relation to product integrity. Operators perceived the climate components communication (Q7) and commitment (Q8) regarding the process integrity better than managers.

These results suggest that human perceptions regarding food integrity might partly be shared within a company across hierarchical levels. The influence of the food integrity climate on organizational functioning depends on the food integrity climate strength, which can be considered as a reflection of the extent of “sharedness” of food integrity climate perceptions among employees [55].

Finally, based on the statistical results obtained from the Cronbach’s alpha analysis, it could be deduced that the overall reliability and internal consistency of the FIC tool is high, with an alpha value of 0.89, which is above the common minimum requirement of 0.7 [56]. Internal consistency of the four subscales, corresponding to the four food integrity elements separately, revealed lower but still acceptable alpha values especially for the product and process integrity scales. In both cases, diminished internal consistency could be attributed to the single negatively formulated item used in both scales (respectively, Q2 and Q10). These two items were not deleted nor reformulated, given the small sample size examined as well as in consideration of the expert validation results and the nomological network of both constructs at hand. With integrity being a sensitive topic potentially leading to socially desirable responding, the use of both positive and negative worded statements may help mitigate the acquiescence bias in responding to a self-reported questionnaire [41,57]. Although their use is recommended by psychometrical scholars [40,41,42,43], such reversed items might have confused or may not have been noticed by some participants in this study, compared to the neutral or positive worded statements [58,59]. Prior testing of the understanding of the tool and its indicators in the target sample as well as supervised data collection with the researchers are recommended for future applications to prevent potential drawbacks of these negatively formulated statements.

### 3.6. Limitations and Future Research

The results from this testing and feedback obtained from the participating company suggest that the FIC tool is clear, understandable, and meaningful. Nonetheless, the outcomes of this study should be considered with caution and further verified in practice in larger groups of respondents and in more heterogeneous samples of food organizations.

Future studies may also examine the relationship between an organization’s food integrity climate and its employees’ behavioral integrity or (un)ethical behavior on a large scale.

Finally, it must be remembering that the food integrity climate self-assessment tool estimates primarily subjective perceptions on the food integrity performance level of a food company. To consolidate the statements made in this paper, such limitations will be tackled in future research through method triangulation, in which the food integrity climate questionnaire outputs will be integrated and compared to the results obtained from a key performance indicators audit to verify objectively a company’s compliance to food integrity standards, and from a food fraud vulnerability assessment tool to get a comprehensive evaluation of the relationship between food fraud and food integrity in a food company.

## 4. Conclusions

Overall, although based on a single small-scale testing, the results illustrated that operators and managers converged in their food integrity perceptions. Their perceptions slightly differed regarding the amount and type of resources spent by their company to embrace food integrity. Operators rated the process integrity highest, while managers considered the process integrity as the lowest.

The FIC tool helps food companies throughout the supply chain in stepping beyond the existing technological and managerial food fraud prevention strategies, since it allows for a deeper insight into the human dimension behind a company’s food integrity through the assessment of five climate components in relation to four food integrity elements. The data collected from the tool assist food businesses in identifying and managing strengths and weaknesses regarding their food integrity climate, and improving performances by evolving from a traditional food safety management system towards a more comprehensive and updated food integrity management system in which food integrity is embraced as a systemic-centric approach inclusive of all technical and non-technical aspects throughout the food supply chain.

## Figures and Tables

**Table 1:** Matrix of the twenty food integrity climate self-assessment tool indicators. Indicators are formulated by matching the notions of the four food integrity elements (product, process, people, and data integrity) [8] with those of the five climate components (leadership, communication, commitment, risk awareness, and resources) [16], adapted to assess food integrity. Each of the four food integrity elements is represented through five indicators and all indicators include a practical example to facilitate respondents’ comprehension of the statements. Questions 2, 10, 13, and 17 are formulated negatively to ensure accurate and unbiased responding. Participants are instructed to score each statement on a five-point Likert scale 1→5: strongly disagree, disagree, not agree nor disagree, agree, strongly agree. The four negatively formulated statements are recoded in reverse during data processing (5→1: strongly disagree→strongly agree). High scores reflect a high perceived food integrity climate. Through the food integrity climate self-assessment tool, managers’ and operators’ perceptions of a company’s food integrity level can be analyzed, acknowledging the human dimension involved in the decision-making processes. (See full questionnaire in Appendix A).

		CLIMATE				
		Leadership	Communication	Commitment	Risk Awareness	Resources
FOOD INTEGRITY	Product	Q1. In my company, leaders set clear objectives and goals on how to achieve product integrity (e.g., leaders give precise tasks and deadlines to employees to deliver products as required by industry standards and according to the company’s recipes).	Q2. In my company, there is *unclear* communication with employees on how to achieve product integrity (e.g., employees’ questions about product requirements, composition and recipes are often badly answered).	Q3. In my company, the importance of product integrity is recognized (e.g., leaders and employees’ main priority is to meet high product standards and fulfill customer requirements).	Q4. In my company, leaders and employees are aware of the hazards and threats related to product integrity (e.g., ingredients adulteration or contamination and product counterfeit or imitation are avoided).	Q5. In my company, the necessary resources are available to achieve product integrity (e.g., good selection of suppliers and raw materials, trained staff and sufficient time to work and perform controls are guaranteed).
	Process	Q6. In my company, leaders have clear expectations on how to achieve process integrity (e.g., leaders require and trust employees to perform processes according to instructions and standard operating procedures).	Q7. In my company, there is clear communication with employees on how to achieve process integrity (e.g., employees understand well leaders’ explanations on how to execute and supervise all the steps of product processing).	Q8. In my company, leaders and employees act properly and constructively to solve issues that affect process integrity (e.g., leaders are prepared to face emergencies; employees are ready to correct incidents or non-compliances on the production line).	Q9. In my company, leaders and employees are aware of the hazards and threats related to process integrity (e.g., equipment, production line and processing methods are kept under control).	Q10. In my company, the necessary resources are *not* always available to achieve process integrity (e.g., equipment, replacement parts, workspaces and systems for production and process monitoring are not sufficient or satisfactory).
	People	Q11. In my company, leaders aim to continuously improve people integrity (e.g., leaders motivate, involve and listen to employees’ concerns and suggestions, behave ethically and lead as role models).	Q12. In my company, the importance of people integrity and ethical behavior is communicated (e.g., employees are encouraged to discuss openly and honestly with leaders and colleagues).	Q13. In my company, working to improve people integrity by behaving ethically is *not* recognized or rewarded (e.g., incentives or positive feedback are not given to employees; dishonest behavior is often ignored or tolerated).	Q14. In my company, people integrity is fostered by being careful, alert and attentive to potential hazards and threats (e.g., employees care about each other’s well-being; leaders respect employees’ rights and take customers’ health seriously).	Q15. In my company, sufficient investments are made to improve people integrity (e.g., good working conditions, ethical code of conduct, conflict mediation service, employees’ training are offered; differences between people and diversity are respected).
	Data	Q16. In my company, leaders aim to continuously improve the level of data integrity (e.g., leaders always verify the quality of the data they receive from suppliers and make sure that food products are delivered as promised in the label and advertisements).	Q17. My company communicates *badly* on the importance of data integrity (e.g., employees receive insufficient written guidelines or receive unclear oral directions on how to prepare, verify and record precise product information).	Q18. In my company, leaders are committed to data integrity by setting a good example (e.g., leaders supervise and participate in work activities ensuring that labels match product properties and product information is recorded and provided correctly).	Q19. In my company, leaders and employees have a realistic picture of hazards and threats related to data integrity (e.g., false documents, invalid statistics or figures, irregular certificates and incorrect labeling are avoided).	Q20. In my company, sufficient investments are made to achieve data integrity (e.g., specific instructions and procedures, good tracking and tracing software, product registration databases and data recording programs are available).

**Table 2:** Expert validation results based on the relevance and importance rating of the food integrity climate self-assessment tool indicators.

Indicator	Relevance ^a,b^	Importance ^c,d,e^
Product Integrity		
Q1	12 (12)	2 (1.83) (0.69)
Q2	8 (12)	2 (2.25) (0.92)
Q3	12 (12)	1 (1.33) (0.47)
Q4	12 (12)	1 (1.33) (0.47)
Q5	11 (11)	1 (1.18) (0.39)
Process Integrity		
Q6	10 (11)	1 (1.82) (1.03)
Q7	12 (12)	1 (1.50) (0.65)
Q8	12 (12)	2 (1.75) (0.72)
Q9	12 (12)	2 (2.08) (0.86)
Q10	8 (11)	2 (2.09) (1.00)
People Integrity		
Q11	12 (12)	2 (1.83) (0.69)
Q12	11 (12)	2 (2.17) (0.99)
Q13	10 (11)	2 (1.91) (0.90)
Q14	11 (11)	2 (1.73) (0.75)
Q15	10 (11)	2 (1.91) (0.79)
Data Integrity		
Q16	12 (12)	1 (1.33) (0.62)
Q17	8 (11)	2 (1.82) (0.94)
Q18	10 (12)	2 (2.08) (0.95)
Q19	11 (12)	2 (2.00) (0.71)
Q20	12 (12)	1 (1.33) (0.47)

^a^ Number of experts considering the indicator relevant. ^b^ Total number of respondents for the indicator. ^c^ Median of the importance rating (1→4: very important→not important). ^d^ Mean of the importance rating (1→4: very important→not important). ^e^ Standard deviation of the importance rating (1→4: very important→not important).

## Appendix A

**Food Integrity Climate Questionnaire**

Thanks for agreeing to participate in our research. This questionnaire investigates the food integrity climate in your company. Responses are completely anonymous and will be treated confidentially.

Food integrity refers to the status of a food product to be intact, safe, of quality, nutritional, authentic in its claims, as well as sourced, produced, and distributed ethically.

Please read the 20 following statements and indicate each time whether you strongly disagree, disagree, not agree nor disagree, agree, or strongly agree by circling the number that represents your answer.

**PRODUCT INTEGRITY** ***Product integrity means that a food product is safe for human consumption, of quality, genuine, with pure and non-adulterated ingredients from recognized and true origins (e.g., product components are not substituted or diluted with cheaper ingredients of lower value or from non-declared origins).***		**Strongly disagree**	**Disagree**	**Not agree nor disagree**	**Agree**	**Strongly agree**
**Q1**	In my company, leaders set clear objectives and goals on how to achieve product integrity (e.g., leaders give precise tasks and deadlines to employees to deliver products as required by industry standards and according to the company’s recipes).	1	2	3	4	5
**Q2**	In my company, there is *unclear* communication with employees on how to achieve product integrity (e.g., employees’ questions about product requirements, composition, and recipes are often badly answered).	1	2	3	4	5
**Q3**	In my company, the importance of product integrity is recognized (e.g., leaders and employees’ main priority is to meet high product standards and fulfill customer requirements).	1	2	3	4	5
**Q4**	In my company, leaders and employees are aware of the hazards and threats related to product integrity (e.g., ingredients adulteration or contamination and product counterfeit or imitation are avoided).	1	2	3	4	5
**Q5**	In my company, the necessary resources are available to achieve product integrity (e.g., good selection of suppliers and raw materials, trained staff, and sufficient time to work and perform controls are guaranteed).	1	2	3	4	5
**PROCESS INTEGRITY** ***Process integrity means that a food product is produced using controlled and regular methods of production, where processes are well executed and assurance standards for food, packaging, hygiene, labor, animal and plant health are followed.***		**Strongly disagree**	**Disagree**	**Not agree nor disagree**	**Agree**	**Strongly agree**
**Q6**	In my company, leaders have clear expectations on how to achieve process integrity (e.g., leaders require and trust employees to perform processes according to instructions and standard operating procedures).	1	2	3	4	5
**Q7**	In my company, there is clear communication with employees on how to achieve process integrity (e.g., employees understand well leaders’ explanations on how to execute and supervise all the steps of product processing).	1	2	3	4	5
**Q8**	In my company, leaders and employees act properly and constructively to solve issues that affect process integrity (e.g., leaders are prepared to face emergencies; employees are ready to correct incidents or non-compliances on the production line).	1	2	3	4	5
**Q9**	In my company, leaders and employees are aware of the hazards and threats related to process integrity (e.g., equipment, production line, and processing methods are kept under control).	1	2	3	4	5
**Q10**	In my company, the necessary resources are *not* always available to achieve process integrity (e.g., equipment, replacement parts, workspaces, and systems for production and process monitoring are not sufficient or inappropriate).	1	2	3	4	5
**PEOPLE INTEGRITY** ***People integrity means that the people working in a company behave honestly and have ethical beliefs and attitudes (e.g., leaders and employees carefully respect rules and comply with high standards).***		**Strongly disagree**	**Disagree**	**Not agree nor disagree**	**Agree**	**Strongly agree**
**Q11**	In my company, leaders aim to continuously improve people’s integrity (e.g., leaders motivate, involve and listen to employees’ concerns and suggestions, behave ethically, and lead as role models).	1	2	3	4	5
**Q12**	In my company, the importance of people integrity and ethical behavior is communicated (e.g., employees are encouraged to discuss openly and honestly with leaders and colleagues).	1	2	3	4	5
**Q13**	In my company, working to improve people’s integrity by behaving ethically is *not* recognized or rewarded (e.g., incentives or positive feedback are not given to employees; dishonest behavior is often ignored or tolerated).	1	2	3	4	5
**Q14**	In my company, people’s integrity is fostered by being careful, alert, and attentive to potential hazards and threats (e.g., employees care about each other’s well-being; leaders respect employees’ rights and take customers’ health seriously).	1	2	3	4	5
**Q15**	In my company, sufficient investments are made to improve people’s integrity (e.g., good working conditions, ethical code of conduct, conflict mediation service, employees’ training are offered; differences between people and diversity are respected).	1	2	3	4	5
**DATA INTEGRITY** ***Data integrity means that all the information regarding a food product is true and accurate, including regular health certifications, valid import documents, and correct product labeling, such as ingredient list, nutritional and technical specifications, origins, and expiry dates.***		**Strongly disagree**	**Disagree**	**Not agree nor disagree**	**Agree**	**Strongly agree**
**Q16**	In my company, leaders aim to continuously improve the level of data integrity (e.g., leaders always verify the quality of the data they receive from suppliers and make sure that food products are delivered as promised in the label and advertisements).	1	2	3	4	5
**Q17**	My company communicates *badly* on the importance of data integrity (e.g., employees receive insufficient written guidelines or receive unclear oral directions on how to prepare, verify and record precise product information).	1	2	3	4	5
**Q18**	In my company, leaders are committed to data integrity by setting a good example (e.g., leaders supervise and participate in work activities ensuring that labels match product properties and product information is recorded and provided correctly).	1	2	3	4	5
**Q19**	In my company, leaders and employees have a realistic picture of hazards and threats related to data integrity (e.g., false documents, invalid statistics or figures, irregular certificates, and incorrect labeling are avoided).	1	2	3	4	5
**Q20**	In my company, sufficient investments are made to achieve data integrity (e.g., specific instructions and procedures, good tracking and tracing software, product registration databases, and data recording programs are available).	1	2	3	4	5

This is the end of the questionnaire. Again, a sincere thank you for your time and collaboration!

## Appendix B

**Table A1:** Overview of mean scores (overall, dimension-specific, and indicator-specific) and student t-test results of the food integrity climate as perceived by managers (*n* = 18) and operators (*n* = 34) of the large-scale meat distribution company participating in the testing of the FIC tool.

	Management	Operators	Student *t*-Test
	Mean	Mean	Significance (*)
Food integrity climate	87.22/100	86.47/100	0.761
Product integrity	22.39/25	21.94/25	0.488
Q1	4.61/5	4.71/5	0.498
Q2	4.56/5	3.94/5	0.015 *
Q3	4.61/5	4.62/5	0.967
Q4	4.28/5	4.44/5	0.500
Q5	4.33/5	4.24/5	0.615
Process integrity	21.39/25	22.03/25	0.378
Q6	4.39/5	4.47/5	0.608
Q7	4.11/5	4.53/5	0.045 *
Q8	4.17/5	4.59/5	0.010 *
Q9	4.39/5	4.56/5	0.317
Q10	4.33/5	3.88/5	0.202
People integrity	21.89/25	20.82/25	0.153
Q11	4.33/5	4.26/5	0.715
Q12	4.39/5	4.18/5	0.212
Q13	4.06/5	3.88/5	0.572
Q14	4.56/5	4.47/5	0.596
Q15	4.56/5	4.03/5	0.008 *
Data integrity	21.56/25	21.68/25	0.866
Q16	4.22/5	4.35/5	0.528
Q17	4.00/5	4.35/5	0.147
Q18	4.44/5	4.35/5	0.624
Q19	4.22/5	4.35/5	0.415
Q20	4.67/5	4.26/5	0.028 *

* *p* < 0.05.
